# Sedative Drug Use among King Saud University Medical Students: A Cross-Sectional Sampling Study

**DOI:** 10.1155/2014/378738

**Published:** 2014-01-14

**Authors:** Ahmed A. Al-Sayed, Abdualltef H. Al-Rashoudi, Abdulrhman A. Al-Eisa, Abdullah M. Addar, Abdullah H. Al-Hargan, Albaraa A. Al-Jerian, Abdullah A. Al-Omair, Ahmed I. Al-Sheddi, Hussam I. Al-Nowaiser, Omar A. Al-Kathiri, Abdullah H. Al-Hassan

**Affiliations:** Department of Community and Family Medicine, College of Medicine, King Saud University, P.O. Box 50652, Riyadh 11533, Saudi Arabia

## Abstract

*Introduction.* Medical students experience significant psychological stress and are therefore at higher risk of using sedatives. There are currently no studies describing the prevalence of sedative drug use among medical students in Saudi Arabia. The aim of this study was to evaluate the prevalence and factors associated with sedative drug use among medical students in Saudi Arabia.* Materials and Methods.* A cross-sectional convenience sampling study gathered data by anonymous questionnaire from students enrolled at the King Saud University College of Medicine in 2011. The questionnaires collected data regarding social and demographic variables, sleep patterns, and the use of stimulant and sedative drugs since enrollment. Sedatives were defined as any pharmaceutical preparations that induce sleep. *Results and Discussion.* Of the 729 students who returned questionnaires, 17.0% reported sedative drug use at some time since enrollment. Higher academic year, lower grade point average, regular exercise, fewer hours of sleep per day, poorer quality of sleep, and the presence of sleeping disorders were found to be significantly associated with sedative drug use. * Conclusions. *Further study is required to increase our understanding of sedative drug use patterns in this relatively high-risk group, as such understanding will help in the development of early intervention programs.

## 1. Introduction

The evidence base linking high rates of stress in students in general [1] and medical students in particular [2] is virtually unassailable. This is from both objective [3] and subjective [4] viewpoints. However, it is unclear if there is a significant level of geographical variation in these rates and a lack of data from Saudi Arabia in particular is notable [5]. As a result it is less clear if stress is a significant issue in Saudi medical students, as with other nations, and therefore coping mechanisms in this population are underexplored [5].

Indeed, a literature search reveals no previous studies linking Saudi medical students, stress levels, and coping mechanisms, although one can note the Pani paper [5], which attempted to quantify stress levels in Saudi dental students and concluded that perceived levels of stress did not always correspond with measured biological levels of stress in this group. However, only forty students participated in this study and the authors explored neither psychological aspects nor coping mechanisms associated with stress.

Psychological manifestations of stress include conditions such as anxiety, [6] depression, [7, 8] or burnout [9]. Students may turn to a number of mechanisms, including cognitive responses, stress management techniques, improved assertiveness skills, time management strategies, and counseling sessions, in order to manage stress [10]. However, inappropriate responses may also occur, including an increased risk of sedative drug use, in an attempt to decrease the symptoms of stress or to deal with resulting mental health issues including sleep [11].

Drug use by medical students is a sensitive and important issue for both the medical profession and society as a whole. Society entrusts health professionals with the administration of prescription medications, and there is concern when they misuse sedative drugs, which can potentially affect their health and performance and could cause addiction or dependence. Sedative drug use among medical students can interfere with their ability to concentrate, as well as causing excessive sleepiness, sluggishness, giddiness, and poor physical coordination [12].

Drug use by medical students has been studied in many countries. A study in the United States reported that 78% of medical students had used psychoactive drugs at some time in their lives [13], although a critical analysis of the study would have to note that this included stimulant drugs as well as sedatives and was not specifically related to medical school stress. A Turkish study found that 22% of junior students, 20% of senior students, and 9% percent of residents used sedative drugs, although this was not exclusively for stress relief, as it included legitimate antiallergic prescribed medication [14]. A study in the Republic of Macedonia found that over 50% of students had used alcohol, specifically to relieve stress, and 12% had used hypnotics [15]. Other studies conducted in Brazil, Nigeria, Vietnam, Norway, Canada, the United States, and other countries reported similar results [16–26]. It would appear therefore that the use of sedative drugs is comparatively widespread across the world in the medical student population. However, no studies to date have reported on the use of these drugs among medical students in Saudi Arabia. Furthermore, the constitutional laws banning alcohol and the tight control over the accessibility of other sedatives in the kingdom makes further studies more difficult.

This study aimed to investigate the prevalence and factors associated with sedative drug use among medical students in Saudi Arabia, as well as the types of sedatives being used in lights of the aforementioned issues.

In the context of this study, one can consider the exemplary review paper by Montgomery et al. [27], which recently reviewed 27 studies concerning self-medication of medical students and newly qualified medical practitioners across a variety of geographical locations and cultures. The authors point out that self-diagnosis and treatment are commonly found in this group, which has potentially severe implications for their own health, both physical and mental, and the quality of care subsequently delivered to patients. Although the scope of the Montgomery paper is very wide, our review will confine comment to the portion related to the use of sedative drugs. The Montgomery paper identifies that it is not simply the need to relieve stress, which is the sole driver to self-medicate with sedative drugs. Four other elements have also been identified as significant. These include avoiding the role of the patient, acceptance of self-treatment as the norm (for various reasons, the alternative, going to a doctor, is less acceptable and less attractive), work performance or pressure to remain at work, and protecting or keeping things within the control of the individual professional or a small number of chosen colleagues (in an effort to retain privacy of health issues).

A carefully constructed study by Roberts et al. [28] explored these issues further and pointed to the fact that medical students had idiosyncratic healthcare patterns. They were considered to generally be challenging and difficult patients as they had issues about confidentiality and also placing themselves in the role of patients in their training institution. Hem et al. [29] found that Norwegian medical students believed that their academic standing would suffer if they were known to develop certain mental health issues, specifically anxiety and depression. All of these factors may well be relevant in the clandestine (unprescribed) use of sedative medication in the target group.

There are also potential long-term consequences of self-medication with sedative drugs as a medical student. The Rosvold and Bjertness study [30] found that Norwegian physicians 9 years after qualification were at greater risk of self-medication with psychoactive drugs if they had used such drugs as medical students. They also found that other risk factors, which included being male, having somatic complaints, experiencing mental distress, suffering from subjective health complaints, and not having sought help from professional colleagues, were also significant predictors of self-medication.

Although a large proportion of research in this field appears to be on Norwegian healthcare professionals, the phenomenon of self-medication in healthcare professionals, even as medical students, appears to be widespread. Papers from USA [31], Australia [32], and Finland [33] all have essentially the same findings.

Uallachain [34] makes the pertinent observation that, even as medical students, there is a widely accepted culture that it is necessary to portray good health and by, default, project competence, by work attendance and having a strong work ethic. This may be instrumental in facilitating the practice of clandestine sedative drug use in medical students who are suffering subjective or objective symptoms of a high workload.

A further element which may be relevant to sedative drug use in medical students is raised by Bennett and O'Donovan [35] who point out that sedative drug use may be considered a means to both maintain work performance and avoid sick leave, with the latter being detrimental to keeping up with the expected workload.

Because of the paucity of studies specifically relating to sedative drug use in medical students, rather than all forms of self-medication per se, it is virtually impossible to get any significant indication of the degree of use. Part of the problem relates to the likely high degree of underreporting from the target group. An indication of the likely size of the problem may be extrapolated from other related studies involving both medical students and newly qualified physicians. Roberts et al. [28] found that 15.7% self-medicated for depression, whereas Evans et al. [31] found that 11.4% used unprescribed benzodiazepines in the preceding year. Rosvold and Bjertness [30] reported a 75% use of benzodiazepines in first-year residents and Hughes et al. [36] reported a 19% use of sedative drugs in US medical students and newly qualified physicians.

There are no studies found which give any indication of the size of the problem in Saudi students. This therefore represents a major gap in the literary evidence base.

## 2. Materials and Methods

A cross-sectional convenience sampling study was undertaken at the King Saud University College of Medicine during 2011. To be eligible to participate in the study, participants were required to be medical students attending the College of Medicine at King Saud University, enrolled and regularly attending their designated courses (first- to fifth-year). Volunteers distributed questionnaires randomly to 744 of the total 1770 students enrolled in the 1st to 5th year courses during March 2011. Students were enrolled in the study at the time of lectures near the end of the academic year, with all students present at those lectures being given the opportunity to participate and were handed a questionnaire. Students were informed that participation was voluntary and anonymous and that information would be kept strictly confidential. The purpose of the study was explained and the students were given an opportunity to ask questions. The participation rate was 41% (729 students out of 1770 participated in the study). The study complied with all institutional ethical requirements and was approved by the Institutional Review Board at the King Saud University College of Medicine. The Review Board also granted permission for publication of the study.

### 2.1. Questionnaire

The questionnaire (see Supplementary Material available online at http://dx.doi.org/10.1155/2014/378738) was developed by the authors and was based on the Montgomery County Court Substance Abuse Questionnaire. The self-administered questionnaire consisted of 25 questions, which collected data regarding demographic, socioeconomic, and lifestyle factors. Students were asked to indicate if they had used any sedative drugs since being enrolled at the College of Medicine, the duration and pattern of use, which drugs they had used, and whether the drugs had been prescribed by a doctor. Sedative drugs were defined as any pharmaceutical preparations that induce sleep.

### 2.2. Statistical Analysis

Students were divided into two groups: sedative drug users (students who had used sedative drugs at any time since enrollment at the College of Medicine) and nonusers (students who had not used any sedative drugs since enrollment). Demographic, socioeconomic, and lifestyle factors were expressed as percentages in both sedative drug user and nonuser groups, and differences between the two groups were compared using the chi-square test or Fisher's exact test, as appropriate. Statistical analyses were performed using Statistical Package for the Social Sciences Software (SPSS) version 19 (SPSS Inc., Chicago, IL, USA). A *P* value of <0.05 was considered statistically significant.

## 3. Results and Discussion

Of the 744 questionnaires distributed, 729 (98%) were returned and analyzed. The respondents included 371 (51%) males and 358 (49%) females, with a mean age (± standard deviation) of 21.1 ± 1.4 years. Of these, 124 students (17.0%) were defined as sedative drug users (63 males, 61 females). Respondents were distributed over different academic years, with 308 (42.2%) students in the 1st and 2nd years (preclinical), 208 (28.5%) students in the 3rd year (mixed preclinical and clinical), and 213 (29.3%) students in the 4th and 5th years (clinical). Almost all respondents who reported using sedative drugs used first-generation antihistamines to induce sleep. Antihistamines were prescribed to 73 students (10.5%) who reported usage, while the remaining 656 students (89.5%) used them without a prescription.

### 3.1. Factors Associated with Sedative Drug Use

Analysis of the results identified significant associations between sedative drug use and academic year, grade point average (GPA), regular exercise, hours of sleep per day, quality of sleep, and sleep disorders (Table 1). Sedative drug use was less common among first- and second-year students than third-year students (12.0% versus 21.6%, odds ratio [OR]: 0.49, 95% confidence interval [CI]: 0.30–0.79, *P* = 0.003). Compared with sedative drug use of 10.7% among students with a GPA of 4.5–5, sedative drug use was more common among students with a lower GPA: 19.5% among students with a GPA of 4–4.49 (OR 2.02, 95% CI: 1.19–3.42, *P* = 0.008), 23.4% among students with a GPA of 3.5–3.99 (OR 2.55, 95% CI: 1.49–4.37, *P* < 0.0001), and 21.1% among students with a GPA of <3.5 (OR 2.23, 95% CI: 1.12–4.47, *P* = 0.02). Sedative drug use was more common among students who exercised regularly than those who did not exercise regularly (26.6% versus 14.6%, OR: 1.68, 95% CI: 1.13–2.51, *P* = 0.01). Sedative drug use was also more common among students who slept for 4–6 hours per day than those who slept for 6–8 hours per day (24.2% versus 15.5%, OR: 1.74, 95% CI: 1.12–2.70, *P* = 0.013) and among students who described their quality of sleep as poor than those who described their quality of sleep as good or excellent (28.8% versus 14.6%, OR: 2.36, 95% CI: 1.49–3.72, *P* < 0.001). Sedative drug use was less common among students who did not report sleeping disorders than those who reported sleeping disorders (10.4% versus 28.8%, OR: 0.28, 95% CI: 0.19–0.43, *P* < 0.001). No significant associations were found between sedative drug use and gender, marital status, parents' education, family income, place of residence, smoking, stimulant use during examination or nonexamination times, or sleep pattern.

### 3.2. Study Implications

The use of various sedative substances among medical students varies between countries depending on differences in culture and availability. To the best of our knowledge, this is the first study reporting the frequency of sedative drug use among medical students in Saudi Arabia. The self-reported rate of sedative drug use was 17.0%, with almost all students who reported using sedatives using antihistamines to induce sleep. This pattern of sedative drug use reflects the lack of availability of alcohol and other sedative drugs in Saudi Arabia, although the overall rate of use seems to be consistent with other international data [14–26]. Alcohol is prohibited by law and is therefore unavailable, and there are tight legal restrictions to the use of other sedative drugs. Benzodiazepines and barbiturates are only available as sleeping aids in tertiary care hospitals and are not prescribed for this use in nonhospitalized patients. Prescription of these drugs for other reasons is carefully controlled, including prescriptions for psychiatric patients, which are only commenced during hospital admission. However, first-generation antihistamines are easily available as over-the-counter medications, and many people use them as sleeping aids [37].

Improved understanding of local patterns of sedative drug use can help in the development of improved education and counseling about the hazards of these drugs. As medical students may experience significant stress, which may result in the use of sedative drugs [7, 9, 11], counseling and preventive mental health should be an integral part of medical school. A previous study of medical students in Saudi Arabia found that 66.6% of female students and 44.4% of male students experienced anxiety or depression [38], which was high compared with an overall prevalence of 26% among primary care patients in Saudi Arabia [39]. Addressing the stressors of medical education and the student environment appears to decrease drug use in future physicians and improve abstinence rates [40].

Sedative drug use was found to be more common among students who exercised regularly than those who did not. This appears to be a paradoxical association, as individuals who exercise regularly would seem to be better at organizing their time including their sleep-wake cycle. This association may be explained by students who are having difficulty sleeping using exercise as a method to promote sleep, together with using other methods such as sedative drugs at times when exercise is not feasible, such as during examination periods.

Sedative drug use was found to be more common among students with a lower GPA. Students with a poorer academic record may have had problems organizing their time, and may therefore have been more likely to use a quick method of inducing sleep at the desired time. The first- and second-year courses are preclinical and may consist of a schedule similar to courses in high school. Starting from the 3rd year, students have a clinical component in their courses, which increases their level of stress and may have caused a higher prevalence of sedative drug use. This is consistent with a study of Nigerian medical students, which found that the prevalence of drug use increased as students advanced through their training [41]. However, the finding that a lower GPA is a risk factor for sedative use is a novel result in the medical student population and further studies are warranted to explore this association in greater detail.

Sedative drug use was found to be more common among students who slept 4–6 hours per day than those who slept 6–8 hours per day. Students who did not get many hours of sleep may not have been as good at organizing their time and their sleep-wake cycles and may therefore have been more likely to use sedative drugs to induce sleep. Students reporting sleep disorders such as insomnia were more likely to use sedative drugs as would be expected. Similarly, there was a higher incidence of sedative drug use among students who described their quality of sleep as poor compared with those who described their quality of sleep as good or excellent.

No significant differences were found between sedative drug users and nonusers for many of the potential associated factors evaluated in this study. This may be partly due to the relatively small sample size and the broad definition of a sedative drug user, which included any student who had used a sedative drug at least once since the beginning of their enrollment at college.

This study was limited by the convenience sampling method used, which may not be representative of the medical school student population. The accuracy of responses on the self-reporting questionnaires may have been adversely affected by the respondents' inability or unwillingness to provide the requested information. If the respondents perceived their sedative drug use to be potentially embarrassing or damaging to their reputation, their answers may not have been truthful, even though confidentiality was assured. There is also the possibility of cognitive distortion or denial on the part of the respondents.

It is difficult to directly compare the use of sedative drugs in the population we studied with other population groups, because of cultural differences in both drug availability and drug use between Saudi Arabia and other countries and the lack of reports describing drug use in the general population of Saudi Arabia. Further studies with a larger sample size and more specific information about the patterns of sedative drug use are required to clarify any associations.

## 4. Conclusions 

17.0% of students reported sedative drug use at some time since enrollment at the College of Medicine. Higher academic year, lower GPA, regular exercise, fewer hours of sleep per day, and the presence of sleeping disorders were significantly associated with sedative drug use. Further understanding of sedative drug use and the factors associated with increased use are helpful for the development of early intervention programs for medical students, who are a high-risk population facing a stressful future career path.

## Supplementary Material

The questionnaire was developed by the authors and collected data regarding social and demographic variables, sleep patterns, and the use of stimulant and sedative drugs among currently enrolled medical students.

## Figures and Tables

**Table 1:** Demographic, socioeconomic, and lifestyle characteristics of sedative drug users and nonusers.

Characteristics	Sedative use, *n* (%)		Total	OR	95% CI	*P* value
	Yes	No				
Gender						
Female*	61 (17.0)	297 (82.9)	358	1		
Male	63 (17.0)	308 (83.0)	371	0.996	0.677–1.466	0.983
Academic year						
3rd*	45 (21.6)	163 (78.4)	208	1		
1st, 2nd	37 (12.0)	271 (88.0)	308	0.495	0.307–0.796	0.003
4th, 5th	42 (19.6)	172 (80.4)	214	0.884	0.552–1.418	0.610
GPA						
4.5–5.0*	28 (10.7)	234 (89.3)	262	1		
4.0–4.49	39 (19.5)	161 (80.5)	200	2.024	1.197–3.423	0.008
3.5–3.99	37 (23.4)	121 (76.6)	158	2.555	1.493–4.375	<0.0001
<3.5	15 (21.1)	56 (78.8)	71	2.239	1.121–4.47	0.02
Marital status						
Others*	2 (8.0)	23 (92.0)	25	1		
Single	122 (17.3)	582 (82.7)	704	2.411	0.56–10.36	0.172^a^
Father's education						
High*	54 (20.3)	212 (79.7)	266	1		
Low	70 (15.1)	393 (84.9)	463	0.699	0.472–1.035	0.073
Mother's education						
High*	18 (19.6)	74 (80.4)	92	1		
Low	106 (16.8)	524 (83.2)	630	0.832	0.477–1.450	0.515
Family income, SR						
>10,000*	104 (17.2)	499 (82.8)	603	1		
<10,000	14 (16.3)	72 (83.7)	86	0.933	0.507–1.717	0.824
Residence						
Others*	11 (14.3)	66 (85.7)	77	1		
Family	113 (17.5)	513 (79.7)	644	1.277	0.653–2.495	0.474
Smoking						
No*	105 (16.2)	542 (83.8)	647	1		
Yes	19 (22.4)	66 (77.6)	85	1.486	0.856–2.579	0.157
Exercise						
No*	74 (14.6)	432 (85.4)	506	1		
Yes	59 (26.6)	163 (73.4)	222	1.687	1.131–2.517	0.01
Stimulants, no exam						
Yes*	120 (17.7)	559 (82.3)	679	1		
No	4 (7.4)	50 (92.6)	54	0.373	0.132–1.052	0.053
Stimulants, exam						
Yes*	122 (17.6)	572 (82.4)	694	1		
No	2 (5.3)	36 (94.7)	38	0.260	0.062–1.096	0.049
Sleep hours						
6–8*	58 (15.5)	317 (84.5)	375	1		
4–6	44 (24.2)	138 (75.8)	182	1.743	1.122–2.705	0.0013
8–10	17 (12.9)	115 (87.1)	132	0.808	0.452–1.445	0.471
>10	5 (12.5)	35 (87.5)	40	0.781	0.294–2.076	0.619
Sleep pattern						
Night/day*	78 (16.6)	392 (83.4)	470	1		
Night	46 (17.8)	212 (82.2)	258	1.09	0.730–1.628	0.672
Sleep quality						
Others*	90 (14.6)	525 (85.4)	615	1		
Poor	34 (28.8)	84 (71.2)	118	2.361	1.495–3.728	<0.001
Sleep disorder						
Yes*	76 (28.8)	188 (71.2)	264	1		
No	46 (10.4)	395 (89.6)	441	0.288	0.192–0.432	<0.001

^a^ *P* value was calculated using Fisher's exact test. All other *P* values are calculated using the chi-square test.

*Reference group.

OR: odds ratio, CI: confidence interval, GPA: grade point average, SR: Saudi Riyal.

OR and CI were calculated using contingency tables or cross-tabulations and logistic regression was used to analyze the associations between the variables.
