# Supplementary material for: Sedative Drug Use among King Saud University Medical Students: A Cross-Sectional Sampling Study
Source: Depress Res Treat. 2014 Jan 14;2014:378738. doi: 10.1155/2014/378738 (PMC3914507; doi:10.1155/2014/378738)
Supplement: Supplementary file 1 — The questionnaire was developed by the authors and collected data regarding social and demographic variables, sleep patterns, and the use of stimulant and sedative drugs among currently enrolled medical students. [file 378738.f1.pdf]

The prevalence of sedative use among KSU's medical students

DISCLOSURE: This questionnaire has been developed independently by the research group and has no corporate interests. The information obtained will be treated with the highest confidentiality measures.

KINDLY ANSWER THE FOLLOWING:

1- Age: ………..

2- Sex: ☐Male ☐Female

3- Class (the first three numbers in your university I.D e.g. 428): …………….

4- Academic year: ☐1st ☐2nd ☐ 3rd ☐ 4th ☐ 5th

5- G.P.A: ☐ 5-4.5 ☐4.5-4 ☐3.5-4 ☐3-3.5 ☐2.5-3 ☐ 2.5 or less

6- Marital status: ☐Single ☐ Married ☐Divorced ☐ Widowed ☐Other, specify: ………….

7- Fathers' level of education: ☐ High school ☐ Bachelors ☐ Masters ☐ PhD ☐ Other, specify ……………

8- Mothers' level of education: ☐High school ☐ Bachelors ☐Masters ☐PhD

☐Other, please specify:………….

9- Family size (number of family members including yourself) …………………………..

10- Average family income per month in S.R:

☐1000-5000 ☐5000-10,000 ☐10,000-15,000 ☐ 15,000-20,000☐ more than 20,000

11- Residence: ☐With family ☐With friends ☐In the dorm ☐Alone ☐Other, specify …………………

12- Are you a smoker (cigarettes, cigars, pipe, Shisha etc...): ☐ Yes ☐No ☐Occasionally ☐ Ex-smoker

13-Do you exercise regularly? (Regular exercise means 20 minutes 3 times a week): ☐Yes ☐No

14- Do you use any type of stimulants including (Coffee ,Tea ,Cola drinks ,Energy Drinks ,etc…)

A-During non-exam periods:

☐ Never ☐Rarely ☐ Occasionally ☐ Always

B- During exam periods:

☐Never ☐ Rarely ☐ Occasionally ☐ Always

15- On average how much time do you spend studying per week? ……………………………….

16- On average how many hours do you sleep per day?

☐4-6 ☐6-8 ☐8-10 ☐more than 10

17- On average how much time do you need to fall asleep? ……………………….

18- Can you specify your sleeping pattern: ☐ Night only ☐Night and day

19- How can you describe your sleep quality?

☐Excellent: uninterrupted, 6-8 hours, deep.
☐Good: wake up once or twice, 5-6 hours, complete rest.
☐Poor: interrupted, less than 5 hours, superficial incomplete rest.

20- Do you experience any of these sleeping disorders?

☐ Insomnia: difficulty initiating or maintaining sleep.

☐ Sleep apnea: obstruction of the airway during sleep.

☐ Restless legs syndrome: An irresistible urge to move legs.

☐ Narcolepsy: falling asleep spontaneously at inappropriate times.

☐ None.

☐ Other, specify …………………………………….

21- Since entering the college of medicine have you ever used sedative drugs e.g. (Panadol Night®, Tylenol pm®, or any other drugs that help you fall asleep): ☐Yes ☐No

If NO, you have finished your questionnaire. If YES please proceed to the following questions:

22- When have you used these drugs?

☐ Regularly ☐ Occasionally ☐Exam periods ☐At the beginning of the school year\term

☐ Other (specify) …………………

23- For how long have you been using these drugs?

☐Isolated occasions ☐Less than 1 year ☐1 – 2 years ☐2 – 3 years ☐more than 3 years

24- Can you please specify the name of the drug: ……………………………….

25- Was the drug prescribed to you by a doctor: ☐Yes ☐No

If Yes, Please specify cause…………………..

THANK YOU FOR COMPLETING THIS SURVEY.

Group (A5) 2010 – 2011, 3rd year medical school, King Saud University.
